# Discordance between Clinical and Ultrasound Examinations in Juvenile Idiopathic Arthritis: An Experimental Approach

**DOI:** 10.3390/children9030333

**Published:** 2022-03-01

**Authors:** Francesco Licciardi, Marco Petraz, Carlotta Covizzi, Francesca Santarelli, Carlotta Cirone, Roberta Mulatero, Francesca Robasto, Marta Dellepiane, Silvana Martino, Davide Montin, Viviana Ravagnani

**Affiliations:** 1 Division of Pediatric Immunology and Rheumatology, Department of Public Health and Pediatrics Sciences, Regina Margherita Children’s Hospital, University of Turin, 10126 Turin, Italy; francesco.licciardi@gmail.com (F.L.); francesca.santarelli86@gmail.com (F.S.); carlotta.cirone@edu.unito.it (C.C.); roberta.mulatero@unito.it (R.M.); francesca.robasto@unito.it (F.R.); martadellepiane@gmail.com (M.D.); silvana.martino@unito.it (S.M.); davide.montin@gmail.com (D.M.); 2 Città della Salute e della Scienza, Department of Radiology, Regina Margherita Children’s Hospital, 10126 Turin, Italy; mpetraz@cittadellasalute.to.it; 3 SSD Allergologia ed Immunologia Clinica, Ospedale C. Poma, ASST Mantova, 46100 Mantova, Italy; viviana.ravagnani@asst-mantova.it

**Keywords:** MSUS, ultrasound, JIA, juvenile idiopathic arthritis

## Abstract

Clinical examination (CE) and musculoskeletal ultrasound (MSUS) of ten joints (knee, ankle, wrist, elbow, II-MCP) and their extra-articular (EA) compartments (tendons and bursae) were performed on 35 consecutive patients with active juvenile idiopathic arthritis (JIA) (active group) to test how the extension of MSUS examinations to EA changes the concordance between MSUS and CE. The overall concordance between CE and MSUS, measured with *Cohen’s Kappa* (*k*), was moderate (k = 0.43); the addition of EA MSUS increased the concordance in all joints, with the exclusion of II-MCP (k = 0.49). In the ankle and wrist, the *k* increase was relevant (k from 0.13 to 0.27 and 0.11 to 0.41). In the active group patients, we observed 44 subclinical synovitis; the number of subclinical synovitis per patient was correlated with JADAS-27 (*p* = 0.03) and was higher in a control group composed of 15 patients with persistent disease remission (1.3 vs. 0.4 *p* = 0.03). Our results show that EA compartments should always be evaluated during MSUS. Furthermore, we demonstrate a moderate concordance between CE and MSUS in JIA; the finding of subclinical synovitis is common in patients with active diseases and is related to disease activity.

## 1. Introduction

In the last decade, musculoskeletal ultrasound (MSUS) have been proposed as useful tools in diagnostic “work-ups” of patients affected by juvenile idiopathic arthritis (JIA). Some authors have suggested that a systematic MSUS evaluation—not only focused on clinically affected joints—might be useful in disease assessment [1]. The application of systematic MSUS has demonstrated a non-optimal concordance between MSUS and clinical examination (CE). This “discordance” may be due to the better sensitivity of MSUS in identifying inflamed joints and to the better ability of MSUS in discriminating the structures. Discordance between MSUS and CE can arise from two different scenarios: joints assessed as inflamed by a CE but negative in the MSUS (CE+/US−) or joints clinically inactive, but with signs of disease activity in a MSUS (subclinical synovitis, CE−/US+). In 2009, Magni-Manzoni studied a cohort of 32 JIA patients (independently of disease status) and found that 18% of CE+ articulations were MSUS− and 51.5% of MSUS+ articulations were CE−. In 2010, Haslam et al. studied 17 patients with oligoarticular JIA and found similar percentages, respectively 26% and 46.9% [2,3]. Unfortunately, these studies were conducted in heterogeneous cohorts and little information is available regarding the concordance in patients in active disease. Two unanswered questions arise from these results: are CE+/US− articulations truly “false positive” or does inflammation interest other periarticular structures, such as the tendon and bursae? Are CE−/US+ joints incidental findings or are they correlated with disease activity? In order to answer these questions, we designed a cohort study enrolling consecutive patients with active JIA and compared them with patients in remission.

## 2. Materials and Methods

### 2.1. Patients

Consecutive patients visited in the outpatient Rheumatology Clinic of Regina Margherita Children Hospital (Turin, Italy) affected by JIA at disease onset or at relapse between April 2017 and April 2018 were enrolled in the study. All patients in the active group had at least 1 active joint and JADAS-27 > 1 [4]. A group of patients with inactive JIA (>6 months), according to JADAS score (JADAS-27 ≤ 1) [4], was used as a control group (remission group).

### 2.2. Clinical Examination and MSUS Evaluation

Patients were first evaluated with routine CE by two experienced pediatric rheumatologists (D.M., S.M.). During examinations, all joints were assessed, joints with swelling or/and tenderness and limited ranges of motion were considered active; in knees, ankles, wrists, and MCPs, the presence of extra-articular swelling suggestive for tendon or bursal involvement was recorded. All patients with active disease who agreed to participate in the study underwent US examinations, which was performed blindly, by clinicians skilled in pediatric MSUS (M.P. or V.R.). Systematic MSUS examinations were performed in five joints: knee, ankle (tibiotalar—TT and subtalar—ST), elbow, wrist (radio–ulno-carpal and inter-carpal), and II metacarpophalangeal (MCP), according to Collado et al. [1]. In each joint, three elements were assessed: synovial hyperplasia, synovial effusion, and the power Doppler signal (pD). The MSUS examiner expressed a binary judgment of the articulation (normal or pathological according to OMERACT indications in children) [5]; in pathological articulations, each of the three elements (hyperplasia, synovial, pD) was expressed with a 0 to 3 grade, according to OMERACT indications in children [5,6], considering age-related vascularization [7].

The following extra-articular structures were assessed during MSUS examinations:

- Tendons: anterior, medial, and lateral ankle compartments; wrist flexor and extensor; II finger extensor and flexor. Common extensor tendon (elbow medial epicondyle), common flexor tendon (elbow lateral epicondyle).
- Bursae: olecranon, prepatellar, pretibial, retrocalcaneal, and retroachilles.

All exams were performed using Esaote Logiq S8 XDclear, with a linear multifrequency array (12–20 MHz).

The US examination technique and the standard scans were based upon OMERACT indications [8].

### 2.3. Concordance Analysis

Concordance between the CE and MSUS was tested firstly, considering the articular compartment alone, after that considering the extra-articular (EA) compartment alone and, finally, considering the articular plus extra-articular compartment (A + EA). Inter-observer concordance was tested firstly with a concomitant, blindly, MSUS of 72 compartments, (blind live concordance), then using still images of 20 patients enrolled in the study (still image concordance); finally intra-observer ex-post concordance was tested, re-analyzing 6 months later the same still images used for inter-observer analysis.

### 2.4. Subclinical Synovitis

The presence of abnormal MSUS findings, consistent with synovitis with normal clinical examination, was defined as *Subclinical synovitis* (CE−/US+). *Clinical synovitis* (CE+/US+) was defined as the concomitant presence of pathological findings at the US and clinical examination. The gradings of CE−/US+ and CE+/US+ in different articulations were compared. Furthermore, correlations between the number of CE−/US+ joints per patient in the active group and demographic variables (sex, age), JIA subsets, ANA positivity, JADAS-27 at the time of the MSUS were tested. Finally, we compared the number of CE−/US+ joints in the active group with the number of CE−/US+ joints in a control group composed of 15 patients with inactive JIA (>6 months), according to the JADAS score [4] (remission group).

### 2.5. Statistical Analysis

For the concordance analysis, *Cohen’s Kappa* (*k*) was used. Concordance was considered poor for k ≤ 0.2, fair for 0.2 < k ≤ 0.4, moderate for 0.4 < k ≤ 0.6, good for 0.6 < k ≤ 0.8, and very good for k > 0.8. Statistical analysis was performed using IBM SPSS Statistics 20.0; GraphPad Prism 6.0. The differences between groups were analyzed using Mann–Whitney U-test for continuous data, or χ^2^ test for categorical data. Correlations between continuous variables were tested using linear regression models. All tests were two-sided and the significance was set at *p* ≤ 0.05.

## 3. Results

### 3.1. Patients

A total of 35 patients with active disease (active group) and 15 patients in clinical remission (remission group) were enrolled in the study. Demographic and clinical data of the active group are summarized in Table 1. Overall, in the active group, active clinical disease was found in 54 articulations: 46.4% knees, 26% ankles (either TT and/or ST), 11% elbows, 9% wrists, and 7.4% II MCP. Extra-articular CE showed 14 tendon involvements in the anterior, medial, or lateral ankle compartment, 2 at II MCP flexor. No extra-articular wrist involvement and no bursitis were reported. Systematic MSUS was performed in all patients, overall, in patients with active disease, 420 articulations, 490 tendineal compartments, and 280 bursae were explored. Pathological MSUS was found in 39 knees, 21 ankles (13 TT and 15 ST), 10 II-MCP, 6 wrists, and 5 elbows (for further details, see Table 2). Regarding MSUS of the extra-articular compartment tenosynovitis was reported in 22 ankles (summing anterior, medial, and lateral compartment), 3 II-MCP (either flexor or extensor tendon), and 2 wrists (either extensor or flexor compartment). Finally, bursitis (3) was found in the pretibial bursa.

### 3.2. Concordance Analysis

Concordance between the CE and MSUS is summarized in Table 3. Overall global articular concordance was moderate (k = 0.43) but ankles and wrists showed a poor concordance (respectively k = 0.13 and k = 0.11). In the ankle, the poor concordance was found, even considering the EA compartment alone (k = 0.12). When concordance was calculated considering A + EA, *k* increased at 0.49. The best performances were achieved in the ankle (k = 0.27) and wrist (k = 0.41). The only compartment where concordance decreased was II-MCP (k = 0.29). Inter-observer variability between MSUS examiners was tested in 72 compartments and was good (k = 0.79), inter-observer variability using still images was very good (k = 0.87), ex-post intra-observer concordance on still images was very good for both observers (k = 0.82 and k = 0.84).

### 3.3. Subclinical Synovitis and Grading

**Figure 1:**
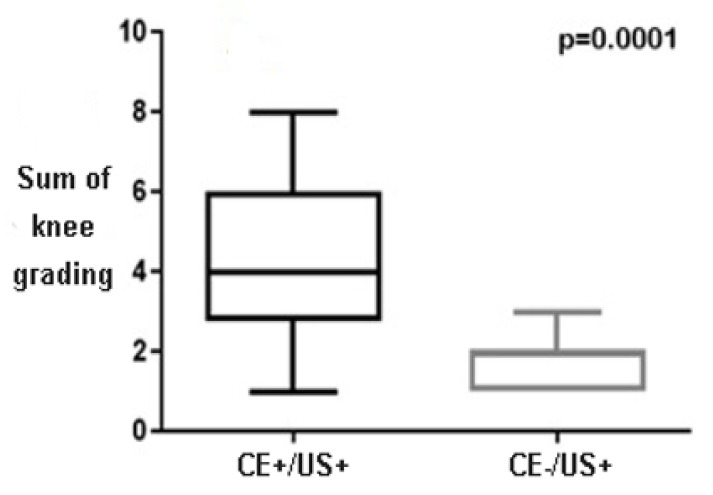
The comparison between the sum of grading (synovial hyperplasia + synovial effusion + PD) in the knee. US, ultrasound; CE, clinical examination.

Overall, CE−/US+ was found in 17 knees, 10 ST, 9 TT and, 7 II-MCP, and 5 wrists in the active group. In the knee, we tested to see if CE−/US+ had a lower MSUS grading in respect to the CE+/US+ joints. In the knee, all three parameters tested were considerably lower in CE−/US+: respectively *p* = 0.008 for synovial hyperplasia (0.9 vs. 1.8), *p* = 0.0005 for synovial effusion (0.6 vs. 1.6), and *p* = 0.01 for PD (0.05 vs. 0.6). The sum of the three gradings in the knee was considerably lower in CE−/US+ (1.6 vs. 4.0 *p* = 0.0001 Figure 1). The small number of MSUS+ ST, TT, wrist, elbow, and II MCP did not allow the grading comparison in these joints.

### 3.4. Subclinical Synovitis and Disease Status

**Figure 2:**
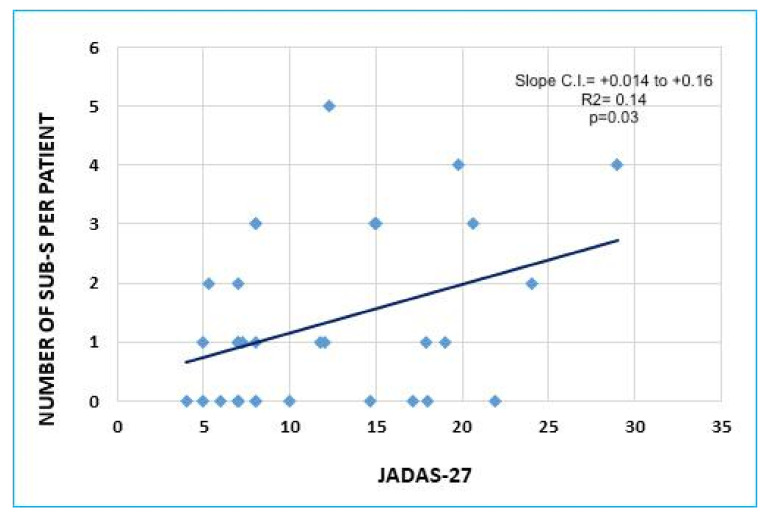
Graphic representation of the linear association between the number of subclinical synovitis (Sub-S: CE−/US+) per patient and JADAS-27 in the active group (*p* = 0.03, R2 = 0.14). C.I., confidence interval.

A higher number of CE−/US+ per patient in the active group was associated with higher JADAS-27 (*p* = 0.03, Figure 2); ANA+ patients had less CE+/US− joints than ANA negative ones (0.8 vs. 1.8 *p* = 0.05). No correlation was found among the number of CE−/US+ joints and age, sex, ESR, and CRP at diagnosis. Clinical data of the remission group are summarized in Table 1. CE−/US+ joints were found in 5 knees and 3 ankles (1 TT and 2 ST). The active group and remission group had overlapping demographic features, except for disease duration (27.7 months vs. 55.7 months *p* = 0.002). Overall, in the remission group, the number of CE−/US+ articulations per patient was considerably lower (0.4 vs. 1.3 *p* = 0.03).

## 4. Discussion and Conclusions

As far as we know, this is the first study of JIA patients with active disease, evaluating the concordance between the MSUS and CE, considering both the intra- and extra-articular compartments in different joints. The study was aimed at this specific cohort because the correct assessment of disease burden in these patients is a crucial step in therapy management. In order to define a protocol used in everyday practice, the MSUS was only addressed at 10 articulations previously selected by Collado et al. as the most informative [1].

The study was designed in order to answer two crucial questions where the use of routine MSUS arise.

1. *Are CE+/US− articulations truly “false positive” or does inflammation interest other periarticular structures, such as tendon and bursae?*

In our cohort, the rate of CE+/US− was 31% of all CE+ articulations, comparable with previously published data. The global *k* showed (due to the concomitant relevant number of CE−/US+ joints) a moderate concordance (k = 0.43). The concordance between the CE and MSUS shifted greatly, depending on the site.

The elbow had the highest concordance (k = 0.90), in this articulation, the CE was sensible and specific in detecting active disease.

The knee showed satisfactory concordance (k = 0.45), in this articulation, the CE seemed specific (12% of “false positive” CE), but the relevant number of CE−/US+ joints decreased the concordance. In this joint, the evaluation of pretibial bursa led to the discovery of bursitis (3), in one of these patients, MSUS of the articulation was normal. This data suggests that evaluation of pretibial bursa should be routinely done in knee MSUS.

Wrists and ankles had the lowest articular *k* (0.11 and 0.13). Studies regarding MSUS performance in ankles have previously been published. In particular, Pascoli et al. showed a poor concordance between the CE and MSUS in TT, and Lanni et al., in ST [9,10]. For this ical examinations, the examiners were only asked to define if the disease interested an articulation (ST or TT) or a tendon. The performance of CE in detecting tendineal involvement was unsatisfactory with poor concordance with a MSUS (k = 0.12). Interestingly, when articular and EA compartments were calculated together, *k* had a relevant increase, reaching 0.27. Our data show that the MSUS is a fundamental tool in the evaluation of this joint. In particular, we confirm that tenosynovitis in this site is very common in JIA and a CE is not able to discriminate between articular and extra-articular involvement [11].

Wrist concordance was never tested before; our results demonstrate that, in this site, the addition of an extra-articular MSUS leads to a significant increase of *k* (from 0.11 to 0.41). In conclusion, EA MSUS should always be performed when evaluating these two articulations, where it explains 44.4% of “false CE positive” joints.

II-MCP is the only site where *k* decreases after an EA compartment analysis; these data may be explained by the presence of subclinical tenosynovitis in some patients. As the clinical significance of these tenosynovitis are still obscure, our data do not suggest routine EA MSUS in this site.

In summary, our data highlight how the concordance between a CE and MSUS varies depending on the joint assessed. As highlighted by other authors, the MSUS and CE evaluate different features of the inflamed joints, leading to a non-optimal concordance [2,11]. Therefore, the CE and MSUS should be considered complementary and not alternative tools in assessing disease status in JIA.

Regarding MSUS, we estimated, for the first time, the impact of routine EA assessments during MSUS in active JIA. As other authors suggest, the involvement of an EA compartment in JIA is much more frequent than previously thought [9,11]. Routine EA assessments increase the global *k* (0.48), and are mandatory, especially during MSUS evaluations of the ankle and wrist.

2. *Is subclinical synovitis (CE−/US+) an incidental finding or is it correlated with disease activity?*

The definition of the role of CE−/US+ is fundamental in order to define if a systematic MSUS (irrespective of CE results) is useful in JIA patient management. Studies regarding the relevance of CE−/US+ in JIA have conflicting results. In particular, Magni Manzoni et al., found no association between subclinical synovitis and functional assessment or acute phase reactants [2]; on the other hand, De Lucia et al., have demonstrated that they predicted disease flare in a group of JIA patients in remission [12]. In our cohort, 54.3% of MSUS synovitis were CE negative; these results overlap with previously mentioned papers. In the active group, we found a significant correlation (*p* = 0.04) between the number of CE−/US+ joints per patient and a widely used disease activity composite score (JADAS-27) [13]. The association was confirmed by the lower number of CE−/US+ in the remission group, despite having significantly longer disease duration. Our results suggest that the CE−/US+ numbers in patients with “active disease” correlates with disease activity, not with disease duration.

Finally, a grading analysis in the knee suggests that, in this joint, subclinical synovitis has very low grading in respect to clinical synovitis, demonstrating that a CE only misses mild/negligible abnormalities in this articulation. Unfortunately, due to the small sample size, we were not able to confirm this finding in other joints.

In conclusion, our results suggest that the number of CE−/US+ joints per patient correlates with disease activity. Prospective studies will be needed in order to define the clinical significance of subclinical synovitis in active JIA.

## Figures and Tables

**Table 1:** Demographic, clinical, and laboratory features, and CE−/US+ in the active and remission groups. ESR, erythrocyte sedimentation rate; CRP, C reactive protein; ANA, anti-nuclear antibodies, CE, clinical examination; US, ultrasound. Of note, disease duration is significantly higher in the remission group while the number of CE−/US+ joints per patient is higher in patients with active disease.

	Active Group	Remission Group	*p*-Value
Total number of patients	35	15	-
Female patients %	80.0%	73.3%	*p* = 0.71
Mean age (months)	102.7 ± 51.5	94 ± 39.5	*p* = 0.43
ILAR category	21 with oligoarthritis (60%); 1 with polyarthritis RF pos (3%) 8 with polyarthritis RF neg (23%) 3 with psoriatic arthritis (8%); 2 with enthesitis-related arthritis (6%).	9 with oligoarthritis (60%); 3 with polyarthritis RF neg (20%); 3 with psoriatic arthritis (20%);	*p* = 1.00
Mean disease duration (months)	27.7 ± 39.5	55.7 ± 37.1	*p* = 0.002
Disease status	16 disease onset (45.7%); 8 relapse *off-therapy* (23%); 11 relapse in MTX (31.4%)	15 disease remission	*p* < 0.0001
ESR (n.v. < 20 mm/h)	20.8 ± 20.5	4.3± 4.8	*p* < 0.0001
CRP (n.v. < 5 mg/L)	7.4 ± 12.7	2.6 ± 2.7	*p* = 0.61
ANA positivity	17 (48.6%)	6 (40.0%)	*p* = 0.76
CE+/US+ per patient	1.0 ±1.2	0	*p* < 0.0001
CE−/US+ per patient	1.3 ± 1.3	0.4 ± 0.7	*p* = 0.03

**Table 2:** Joints with synovitis at clinical examination (CE) and/or at the MSUS (US). Numbers are expressed as % of total joints assessed. US, ultrasound; CE, clinical examination; MCP metacarpophalangeal joint. In bold are expressed significant *p*-values.

		CE+	CE−
ELBOW	US+	7%	0%
	US−	2%	91%
KNEE	US+	31%	24%
	US−	4%	41%
WRIST	US+	1%	7%
	US−	6%	86%
ANKLE	US+	9%	21%
	US−	11%	59%
II MCP	US+	4%	10%
	US−	1%	85%
TOTAL	US+	11%	13%
	US−	5%	71%

**Table 3:** Concordance between physical examinations and MSUS, considering the articular compartment alone and the articular + extra-articular compartment. Concordance was evaluated using Cohen’s Kappa coefficient (k). C.I., confidence interval; MCP metacarpophalangeal joint.

	Articular Compartment	Articular + Extra-Articular Compartment
ELBOW	k = 0.89 C.I. (0.70–1.0)	k = 0.89 C.I. (0.70–1.0)
KNEE	k = 0.45 C.I. (0.26–0.63)	k = 0.48 C.I. (0.27–0.65)
WRIST	k = 0.11 C.I. (0.00–0.43)	k = 0.41 C.I. (0.06–0.76)
ANKLE	k = 0.13 C.I. (0.00–0.38)	k = 0.27 C.I. (0.05–0.49)
II MCP	k = 0.38 C.I. (0.05–0.71)	k = 0.29 C.I. (0.00–0.58)
TOTAL	k = 0.43 C.I. (0.32–0.55)	k = 0.49 C.I. (0.37–0.59)

## Data Availability

The data presented in this study are available on request from the corresponding author. The data are not publicly available due to privacy (data are not anonymized).
